# Rapid and Accurate Molecular Identification of the Emerging Multidrug-Resistant Pathogen Candida auris

**DOI:** 10.1128/JCM.00630-17

**Published:** 2017-07-25

**Authors:** Milena Kordalewska, Yanan Zhao, Shawn R. Lockhart, Anuradha Chowdhary, Indira Berrio, David S. Perlin

**Affiliations:** a Public Health Research Institute, Rutgers Biomedical and Health Sciences, Newark, New Jersey, USA; b Mycotic Diseases Branch, Centers for Disease Control and Prevention, Atlanta, Georgia, USA; c Department of Medical Mycology, Vallabhbhai Patel Chest Institute, University of Delhi, Delhi, India; d Clínica El Rosario, Medellín, Colombia; e Medical and Experimental Mycology Group, Corporación para Investigaciones Biológicas (CIB), Medellín, Colombia; f Hospital General de Medellin Luz Castro de Gutiérrez ESE, Medellín, Colombia; University of Iowa College of Medicine

**Keywords:** Candida duobushaemulonii, Candida haemulonii, Candida lusitaniae, Candida auris, PCR, diagnostics, identification, real-time PCR

## Abstract

Candida auris is an emerging multidrug-resistant fungal pathogen causing nosocomial and invasive infections associated with high mortality. C. auris is commonly misidentified as several different yeast species by commercially available phenotypic identification platforms. Thus, there is an urgent need for a reliable diagnostic method. In this paper, we present fast, robust, easy-to-perform and interpret PCR and real-time PCR assays to identify C. auris and related species: Candida duobushaemulonii, Candida haemulonii, and Candida lusitaniae. Targeting rDNA region nucleotide sequences, primers specific for C. auris only or C. auris and related species were designed. A panel of 140 clinical fungal isolates was used in both PCR and real-time PCR assays followed by electrophoresis or melting temperature analysis, respectively. The identification results from the assays were 100% concordant with DNA sequencing results. These molecular assays overcome the deficiencies of existing phenotypic tests to identify C. auris and related species.

## INTRODUCTION

Candida auris is an emerging multidrug-resistant yeast that can cause invasive infections and is associated with high mortality. It was first described in 2009 after being isolated from the external ear discharge of a patient in Japan (1). Since then, C. auris infections have been reported from South Korea (2, 3), India (4–6), Pakistan (5), Kuwait (7), Israel (8), South Africa (5, 9), the United Kingdom (10–12), Spain (13), the United States (14, 15), Colombia (16), and Venezuela (5, 17). The Centers for Disease Control and Prevention (CDC) and other research groups reported that almost all C. auris isolates are highly resistant to fluconazole, with the other azoles showing variable antifungal activity and isavuconazole and posaconazole being the most active ones. Moreover, up to one-third were resistant to amphotericin B, and a few were resistant to echinocandins. Some isolates demonstrated elevated MICs to all three major antifungal classes (azoles, echinocandins, and polyenes), indicating that treatment options against these multidrug-resistant isolates would be limited (18–20). C. auris is of great concern to public health agencies, due to the possibility that biologic and epidemiologic factors could trigger an even more extensive worldwide emergence of C. auris infections (21). Therefore, it is important for clinical microbiology and public health laboratories to rapidly and accurately identify this organism to help prevent health care-associated outbreaks and improve survival among infected patients by enabling appropriate early antifungal therapy implementation (22, 23).

C. auris is phenotypically close to Candida haemulonii (1). It was reported that laboratories worldwide, relying on commercially available phenotypic platforms for yeast identification, commonly misidentify C. auris as C. haemulonii but also as several other yeast species (C. famata, C. guilliermondii, C. lusitaniae, C. parapsilosis, C. sake, Rhodotorula glutinis, and Saccharomyces cerevisiae) (18, 22, 24, 25). Moreover, some clinical laboratories do not identify all Candida to the species level, placing C. auris isolates in the “other Candida spp.” category (18). Thus, the prevalence of C. auris is probably significantly underestimated due to unreliable identifications (4, 18, 25).

Given the current diagnostic urgency surrounding this pathogen, the aim of this work was to develop molecular-based methods that can quickly and accurately identify C. auris and related species (C. duobushaemulonii, C. haemulonii, and C. lusitaniae). The performance of the proposed methodology was evaluated using a comprehensive panel of clinical isolates with a wide spectrum of variable fungal species.

## RESULTS

### Primer design.

The specific primers enabling the identification of C. auris and related species, C. duobushaemulonii, C. haemulonii, and C. lusitaniae are listed in Table 1. The designed amplicons cover a fragment of 5.8S, all of ITS2, and a fragment of 28S. CauF and CauR primers were designed to selectively amplify a 163-bp-long PCR product specific for C. auris only. CauRelF and CauRelR primers were designed to selectively amplify PCR products from either C. auris, C. duobushaemulonii, C. haemulonii, or C. lusitaniae. Amplified fragments differ in length (215 bp, 208 bp, 197 bp, and 203 bp, respectively) and composition and can be easily distinguished upon melting curve analysis.

**TABLE 1:** Primers used in the study

Primer	Sequence	Specificity
CauF	5′-CGCACATTGCGCCTTGGGGTA-3′	C. auris
CauR	5′-GTAGTCCTACCTGATTTGAGGCGAC-3′	
CauRelF	5′-GCGATACGTAGTATGACTTGCAGACG-3′	C. auris and related species (C. duobushaemulonii, C. haemulonii, and C. lusitaniae)
CauRelR	5′-CAGCGGGTAGTCCTACCTGA-3′	

### Candida auris-specific PCR and real-time PCR assays.

**FIG 1:**
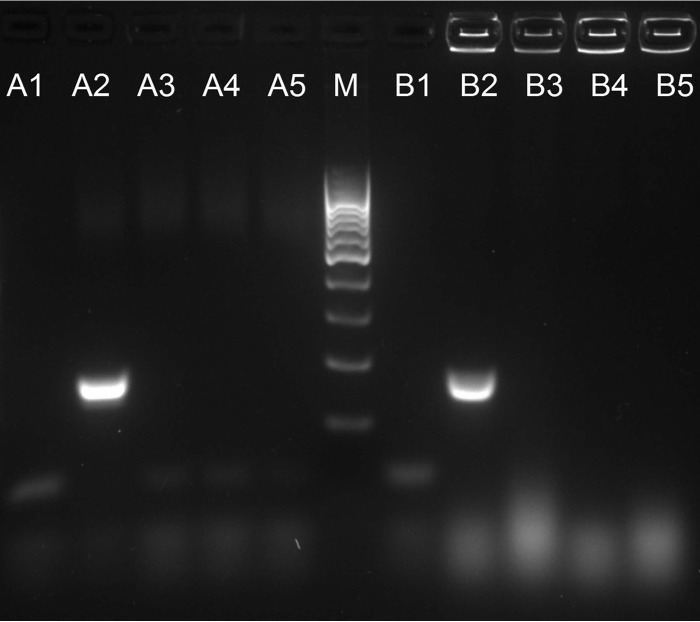
Example of Candida auris-specific PCR product analysis. M, 100-bp DNA ladder (fragment sizes 1,000, 900, 800, 700, 600, 500, 400, 300, 200, and 100 bp); A series, results of Candida auris-specific PCR; B series, results of colony Candida auris-specific PCR; lane 1, negative control; lane 2, C. auris VPCI 671/P/12; lane 3, C. haemulonii ATCC 22991; lane 4, C. duobushaemulonii B09383; lane 5, C. lusitaniae CAS08-0577; VPCI, Vallabhbhai Patel Chest Institute.

A 163-bp PCR product specific for Candida auris was observed for all 44 C. auris DNA samples. No PCR products were detected for other yeast and mold isolates or human DNA (100% sensitivity and 100% specificity). Moreover, robust and reproducible amplicons were observed for all isolates when DNA extracts were replaced with a direct single-colony pick in the established assay (Fig. 1).

**FIG 2:**
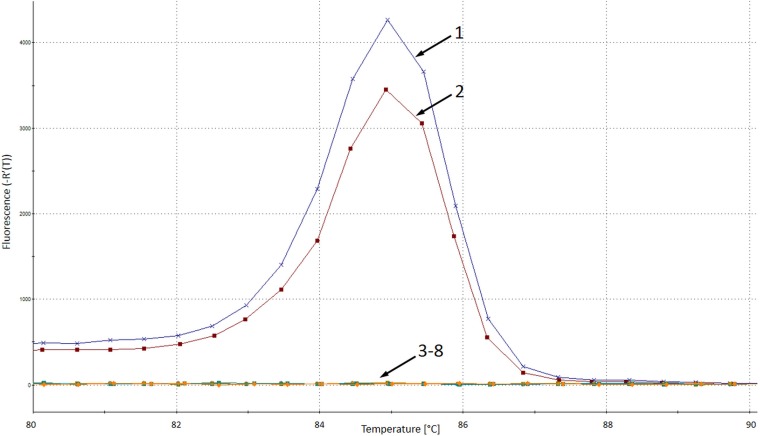
Melting profile of Candida auris-specific real-time PCR. 1, C. auris B11800 (Colombia); 2, C. auris VPCI 1133/P/13 (India); 3, C. duobushaemulonii CAS11-3561; 4, C. lusitaniae DPL 284; 5, C. albicans DPL 225; 6, C. sake 3000724892; 7, Saccharomyces cerevisiae DPL 269; 8, negative control; VPCI, Vallabhbhai Patel Chest Institute; DPL, David Perlin Laboratory.

**FIG 3:**
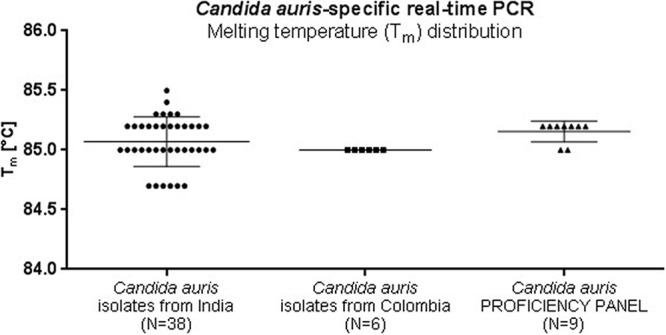
Distribution of melting temperatures of amplicons obtained for C. auris isolates in the Candida auris-specific real-time PCR assay.

Similar results were obtained when real-time PCR was applied, as an amplicon with a melting temperature (*T_m_*) of 85.1 ± 0.2°C, corresponding to C. auris, was observed only for 44 C. auris DNA samples and not for any other fungal or human DNA samples (Fig. 2 and Table 2). The limit of detection (LOD) for the C. auris-specific assay was established at the level of 10 CFU/reaction (threshold cycle [*C_T_*], 28.61 ± 0.25). The accuracy of the assay was confirmed by a proficiency test against a panel of 46 isolates (Table 3). The distribution of the amplicons' melting temperatures obtained for C. auris isolates is presented in Fig. 3.

**TABLE 2:** Specificity of Candida auris-specific and Candida auris related-species-specific assays

Species or organism	Candida auris-specific PCR	Real-time PCR			
		Candida auris-specific		Candida auris related-species-specific	
	No. of isolates detected/no. tested	No. of isolates detected/no. tested	*T_m_* (°C)	No. of isolates detected/no. tested	*T_m_* (°C)
C. auris	44/44	44/44 (*C_T_*, 19.4 ± 2.5)	85.1 ± 0.2	44/44 (*C_T_*, 17.7 ± 1.8)	85.6 ± 0.15
C. haemulonii	0/7	0/7	—^*a*^	7/7 (*C_T_*, 18.2 ± 1.3)	84.8 ± 0.2
C. duobushaemulonii	0/6	0/6	—	6/6 (*C_T_*, 20.4 ± 0.7)	86.2 ± 0.1
C. lusitaniae	0/6	0/6	—	6/6 (*C_T_*, 20.1 ± 0.6)	87.6 ± 0.1
C. albicans	0/9	0/9	—	0/9	—
C. glabrata	0/10	0/10	—	0/10	—
C. tropicalis	0/11	0/11	—	0/11	—
C. krusei	0/10	0/10	—	0/10	—
C. parapsilosis	0/10	0/10	—	0/10	—
C. metapsilosis	0/4	0/4	—	0/4	—
C. orthopsilosis	0/3	0/3	—	0/3	—
C. dubliniensis	0/3	0/3	—	0/3	—
C. guilliermondii	0/4	0/4	—	0/4	—
C. kefyr	0/2	0/2	—	0/2	—
C. famata	0/1	0/1	—	0/1	—
C. sake	0/1	0/1	—	0/1	—
Rhodotorula mucilaginosa	0/3	0/3	—	0/3	—
Saccharomyces cerevisiae	0/2	0/2	—	0/2	—
Aspergillus fumigatus	0/1	0/1	—	0/1	—
A. flavus	0/1	0/1	—	0/1	—
A. niger	0/1	0/1	—	0/1	—
Fusarium solani	0/1	0/1	—	0/1	—
Human	0/1	0/1	—	0/1	—

a no *T_m_*.

**TABLE 3:** Proficiency panel results of Candida auris-specific and Candida auris related-species-specific assays

Species	Candida auris-specific real-time PCR		Candida auris related-species-specific real-time PCR	
	No. of isolates detected/no. tested	*T_m_* (°C)	No. of isolates detected/no. tested	*T_m_* (°C)
C. auris	9/9 (*C_T_*, 18.5 ± 1)	85.1 ± 0.1	9/9 (*C_T_*, 18.3 ± 1.8)	85.8 ± 0.1
C. haemulonii	0/7	—^*a*^	7/7 (*C_T_*, 20.9 ± 0.6)	84.8 ± 0.15
C. duobushaemulonii	0/6	—	6/6 (*C_T_*, 22.4 ± 1.2)	86.2 ± 0
C. lusitaniae	0/6	—	6/6 (*C_T_*, 21.6 ± 1.6)	87.6 ± 0.1
C. albicans	0/2	—	0/2	—
C. glabrata	0/2	—	0/2	—
C. tropicalis	0/2	—	0/2	—
C. krusei	0/2	—	0/2	—
C. parapsilosis	0/2	—	0/2	—
C. dubliniensis	0/1	—	0/1	—
C. guilliermondii	0/1	—	0/1	—
C. kefyr	0/1	—	0/1	—
C. famata	0/1	—	0/1	—
C. sake	0/1	—	0/1	—
Rhodotorula mucilaginosa	0/1	—	0/1	—
Saccharomyces cerevisiae	0/2	—	0/2	—

a no *T_m_*.

### Candida auris related-species-specific real-time PCR assay.

**FIG 4:**
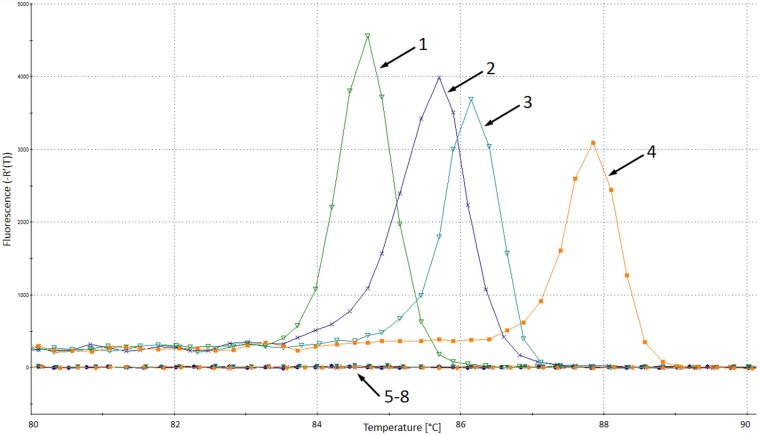
Melting analysis of Candida auris related-species-specific real-time PCR. 1, C. haemulonii ATCC 22991; 2, C. auris VPCI 1133/P/13; 3, C. duobushaemulonii CAS11-3561; 4, C. lusitaniae DPL 284; 5, C. albicans DPL 225; 6, C. sake 3000724892; 7, Saccharomyces cerevisiae DPL 269; 8, negative control; VPCI, Vallabhbhai Patel Chest Institute; DPL, David Perlin Laboratory.

**FIG 5:**
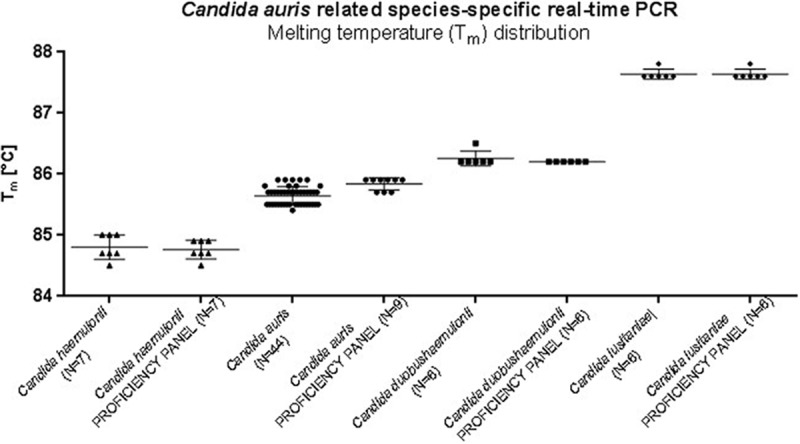
Distribution of melting temperatures of amplicons obtained for C. haemulonii, C. auris, C. duobushaemulonii, and C. lusitaniae isolates in the Candida auris related-species-specific real-time PCR assay.

PCR products were observed for the following DNA samples: 7 C. haemulonii isolates (*T_m_*, 84.8 ± 0.2°C), 44 C. auris isolates (*T_m_*, 85.6 ± 0.15°C), 6 C. duobushaemulonii isolates (*T_m_*, 86.2 ± 0.1°C), and 6 C. lusitaniae isolates (*T_m_*, 87.6 ± 0.1°C). No PCR products were detected for other yeast and mold isolates or human DNA (100% sensitivity and 100% specificity) (Fig. 4 and Table 2). The LOD for the C. auris related species-specific assay was established at the level of 1,000 CFU/reaction (*C_T_*, 27.83 ± 0.87). The accuracy of the assay was confirmed by testing a proficiency panel of 46 isolates (Table 3). The distribution of the amplicons' melting temperatures obtained for C. haemulonii, C. auris, C. duobushaemulonii, and C. lusitaniae isolates is presented in Fig. 5.

## DISCUSSION

In 2016, the CDC released an alert informing of an emerging pathogen, Candida auris, that is causing invasive infections (18), due to the challenging identification, common multidrug resistance, and outbreaks of this pathogen in health care settings (15).

A rapid and accurate identification of C. auris is important for not only the appropriate use of antifungal treatment but also the implementation of effective infection control measures (12, 26). However, many laboratories do not routinely identify non-albicans Candida isolates to the species level, or they utilize phenotype-based yeast identification methods, such as chromogenic agar, biochemical tests, or automated systems, which commonly misidentify C. auris as many different yeast species. C. auris may be misidentified as C. sake, Rhodotorula glutinis, or Saccharomyces cerevisiae by API 20C AUX, as C. haemulonii by BD Phoenix, as C. haemulonii or C. famata by Vitek-2, or as C. famata, C. lusitaniae, C. guillermondii, or C. parapsilosis by MicroScan (12, 18, 22, 24, 25). Nowadays, proper identification of Candida species requires the application of specialized methods such as matrix-assisted laser desorption ionization–time of flight (MALDI-TOF) or molecular identification based on sequencing the D1-D2 region of the 28S ribosomal DNA. However, due to the lack of C. auris entries in the FDA-approved libraries, it remains unidentified by Bruker Biotyper and Vitek-MS, and only when an additional research use only (RUO) library containing C. auris is incorporated can correct identification of this organism be obtained by both MALDI systems (25, 27).

In this study, we addressed the challenging identification of Candida auris. We present both conventional and real-time PCR assays that allow specific identification of C. auris and related species (C. duobushaemulonii, C. haemulonii, and C. lusitaniae) within 2 (real-time PCR and colony PCR) to 2.5 (conventional PCR) hours. To meet different diagnostic needs, we proposed two assays of different specificity ranges; the first assay identifies C. auris only, while in the second assay, C. auris, C. duobushaemulonii, C. haemulonii, and C. lusitaniae can be identified and distinguished from each other. The differential specificities of the assays were obtained by a detailed analysis of rDNA sequences deposited in the NCBI nucleotide database that enabled the design of highly specific primers. Using 140 fungal isolates and human genomic DNA, we were able to identify C. auris isolates with 100% accuracy in all developed assays. Moreover, in C. auris related-species-specific real-time PCR, signature melting profiles and corresponding *T_m_* values were generated for C. auris, C. duobushaemulonii, C. haemulonii, and C. lusitaniae, enabling their unambiguous discrimination. Excellent results were achieved with both assays during the development phase, as well as during the proficiency panel validation.

In summary, we have developed two rapid, accurate, easy-to-perform and interpret molecular diagnostic assays to identify C. auris and related species (C. duobushaemulonii, C. haemulonii, and C. lusitaniae) that overcome the deficiencies of existing phenotypic assays. Moreover, we expect that in the future, this diagnostic platform may be adjusted for the direct detection of C. auris in swabs from patients and from the hospital environment.

## MATERIALS AND METHODS

### Fungal isolates and culture conditions.

In this study, we used a total of 140 fungal isolates (9 C. albicans, 44 C. auris, 3 C. dubliniensis, 6 C. duobushaemulonii, 1 C. famata, 10 C. glabrata, 4 C. guilliermondii, 7 C. haemulonii, 10 C. krusei, 6 C. lusitaniae, 4 C. metapsilosis, 3 C. orthopsilosis, 10 C. parapsilosis, 2 C. kefyr, 1 C. sake, 11 C. tropicalis, 3 Rhodotorula mucilaginosa, 2 Saccharomyces cerevisiae, 1 Aspergillus fumigatus, 1 A. flavus, 1 A. niger, and 1 Fusarium solani) and 1 sample of human genomic DNA (Roche). Thirty-eight C. auris isolates were obtained from Vallabhbhai Patel Chest Institute, University of Delhi (Delhi, India), and 6 isolates were obtained from Clinica General del Norte (Barranquilla, Colombia). The remaining 96 laboratory and clinical isolates were stocked in the Perlin laboratory collection at the Public Health Research Institute (Newark, NJ, USA) (69 isolates) and the Fungal Reference Laboratory Collection at the Centers for Disease Control and Prevention (Atlanta, GA, USA) (27 isolates). Isolates were grown on yeast extract-peptone-dextrose (YPD) agar plates (at 24°C for C. haemulonii, C. duobushaemulonii, and C. sake isolates and at 37°C for all other isolates) prior to testing. Species identification of all Candida isolates was performed by sequencing of the rDNA region (partial sequences of the 18S and 28S rRNA genes and complete sequences of the internal transcribed spacer 1, 5.8S rRNA gene, and internal transcribed spacer 2), which was amplified with Fun-rDNAF (5′-GGTCATTTAGAGGAAGTAAAAGTCG-3′) and Fun-rDNAR (5′-YGATATGCTTAAGTTCAGCGGGTA-3′) primers (S. Katiyar, personal communication), and further nucleotide BLAST analysis (https://blast.ncbi.nlm.nih.gov/Blast.cgi).

### DNA extraction.

In all specificity tests and proficiency panel experiments, DNA from fungal isolates was prepared by a 10-min incubation of a single colony in 100 μl of extraction buffer (60 mM sodium bicarbonate [NaHCO_3_], 250 mM potassium chloride [KCl], and 50 mM Tris, pH 9.5) at 95°C and the subsequent addition of 100 μl anti-inhibition buffer (2% bovine serum albumin). After vortex mixing, this DNA-containing solution was used for PCR (28). As for the analytical sensitivity evaluation experiments, DNA was isolated using a FastDNA kit (MP Biomedicals) according to the manufacturer's instruction.

### Primer design.

According to the rDNA sequence alignment (DNASTAR Lasergene 14), primers specific for Candida auris only or for C. auris and related species (C. duobushaemulonii, C. haemulonii, and C. lusitaniae) were designed (Table 1). Primers were then synthesized by Integrated DNA Technologies.

### Candida auris-specific PCR.

PCR mixtures were prepared in a total volume of 30 μl, consisting of 15 μl of 2× EmeraldAmp MAX PCR master mix (TaKaRa Bio Inc.), 1 μl of each primer (CauF and CauR) at 10 μM, and 2 μl of DNA. PCR was performed in a T100 thermal cycler (Bio-Rad Laboratories, Inc.). The thermal profile included an initial denaturation for 3 min at 95°C followed by 30 cycles of 20 s at 95°C, 20 s at 68°C, and 20 s at 72°C. The presence of amplicons was examined electrophoretically on 2% agarose gels stained with GelStar (Lonza).

### Candida auris-specific and Candida auris related-species-specific real-time PCR.

Species-specific and related-species-specific real-time PCR mixtures were 30 μl per reaction, containing 15 μl of 2× One-Step SYBR RT-PCR buffer IV, 1 µl of PrimeScript enzyme mix II (TaKaRa Bio, Inc.), 1 μl of each primer (CauF and CauR or CauRelF and CauRelR, respectively) at 10 μM, and 2 μl of DNA. Real-time PCR was performed on an Mx3005P qPCR system (Stratagene). The Candida auris-specific assay consisted of a 3-min incubation at 95°C, followed by 30 cycles of 15 s at 95°C, 20 s at 68°C, and 20 s at 72°C, and then 72°C for 5 min. The Candida auris related-species-specific assay consisted of a 3-min incubation at 95°C, followed by 30 cycles of 15 s at 95°C, 30 s at 66°C, and 30 s at 72°C, and then 72°C for 5 min. Immediately after amplification, a melting curve analysis was performed at 95°C for 1 min, and then from 70°C to 95°C with a ramp rate of 0.2°C/s.

### Analytical sensitivity evaluation.

The analytical sensitivity of the assays was determined by testing 10-fold serial dilutions of DNA samples ranging from 1 to 10^6^ CFU/reaction in triplicates. Two C. auris isolates were initially tested for the Candida auris-specific assay, and 1 C. auris, 1 C. duobushaemulonii, 1 C. haemulonii, and 1 C. lusitaniae were used to evaluate the Candida auris related-species-specific assay. The LOD was determined as the smallest amount of template that elicited a positive *C_T_* (threshold cycle) value and an unambiguous melting profile.

### Proficiency panel.

Real-time PCR assay performance was validated on a panel of 46 clinical isolates (2 C. albicans, 9 C. auris, 1 C. dubliniensis, 6 C. duobushaemulonii, 1 C. famata, 2 C. glabrata, 1 C. guilliermondii, 7 C. haemulonii, 2 C. krusei, 6 C. lusitaniae, 2 C. parapsilosis, 1 C. kefyr, 1 C. sake, 2 C. tropicalis, 1 Rhodotorula mucilaginosa, and 2 Saccharomyces cerevisiae) pulled from the initial 140 isolates. The proficiency test was performed by a person who was blind to the sample identification (ID).

### Colony Candida auris-specific PCR.

To further reduce the time to diagnosis, we introduced a colony PCR into our diagnostic assay. Instead of using DNA extracts, a sterile toothpick was touched to a single colony and dipped into the PCR mixture, and then Candida auris-specific PCR was performed as described above. Forty-six clinical isolates pulled from the initial 140 isolates (proficiency panel) were tested for the efficiency of the colony PCR.

### Statistical analysis.

Melting temperature (*T_m_*) values for each species were determined by melting curve analysis using the MxPro software (version 4.1) (Stratagene). The *T_m_* distribution was analyzed by GraphPad Prism 6.05 software. The accuracies of the novel assays discriminating C. auris from other species were evaluated by calculating the sensitivity and specificity for each assay.
